# Supplementary material for: Synergistic Effect of Surface Acidity and PtOx Catalyst on the Sensitivity of Nanosized Metal–Oxide Semiconductors to Benzene
Source: Sensors (Basel). 2022 Aug 29;22(17):6520. doi: 10.3390/s22176520 (PMC9460263; doi:10.3390/s22176520)
Supplement: Supplementary file 1 [file sensors-22-06520-s001.zip › sensors-1885326-supplementary.pdf]

Supplementary data

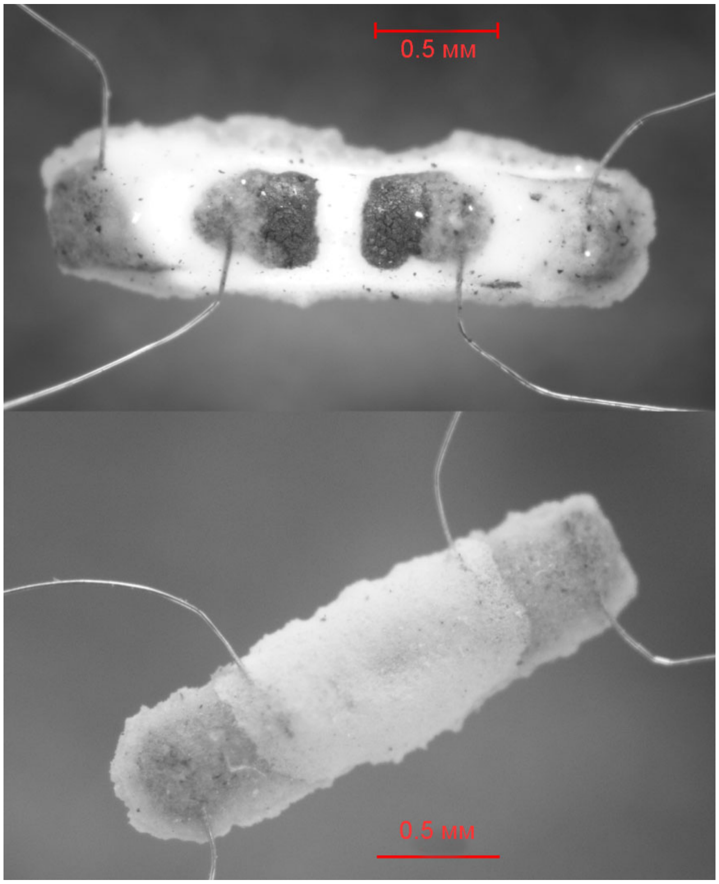

Figure S1. Microscope images of sensor substrate before (top) and after (bottom) thick film deposition.

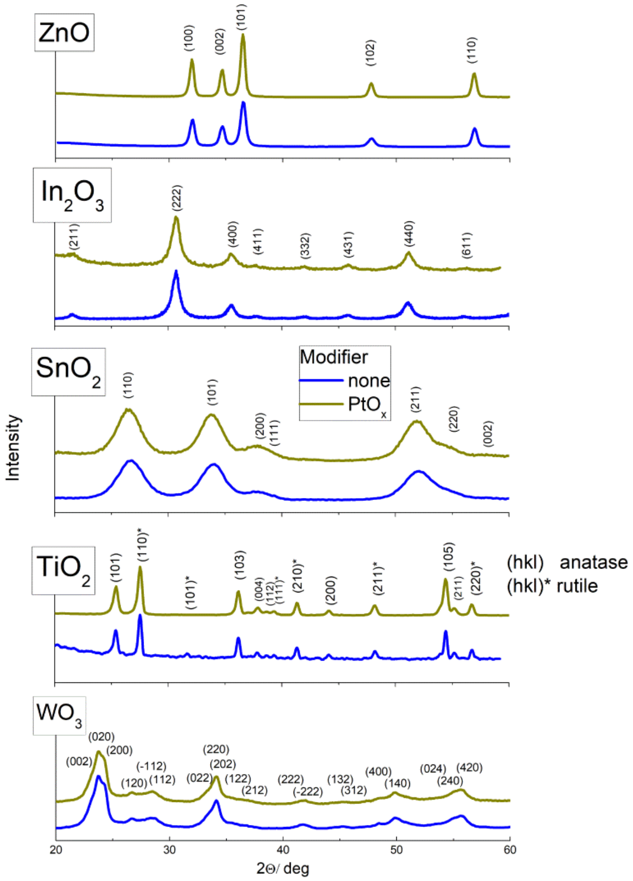

Figure S2. XRD patterns of MOS and MOS/PtO_x samples.

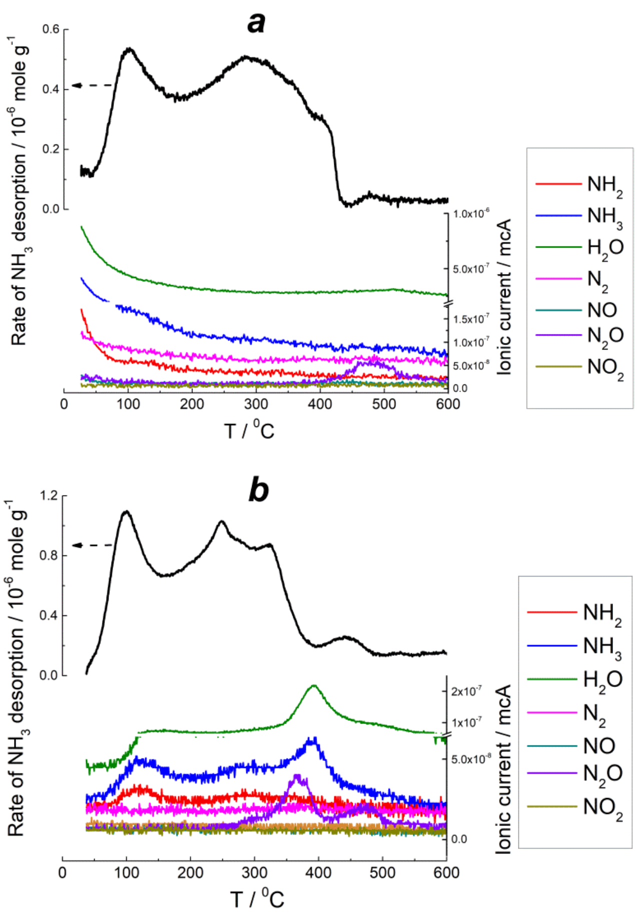

Figure S3. Patterns of ammonia TPD from the surface of MOS (thermal conductivity detector, TCD) and mass-spectrometric analysis (MS) of desorbed gas.

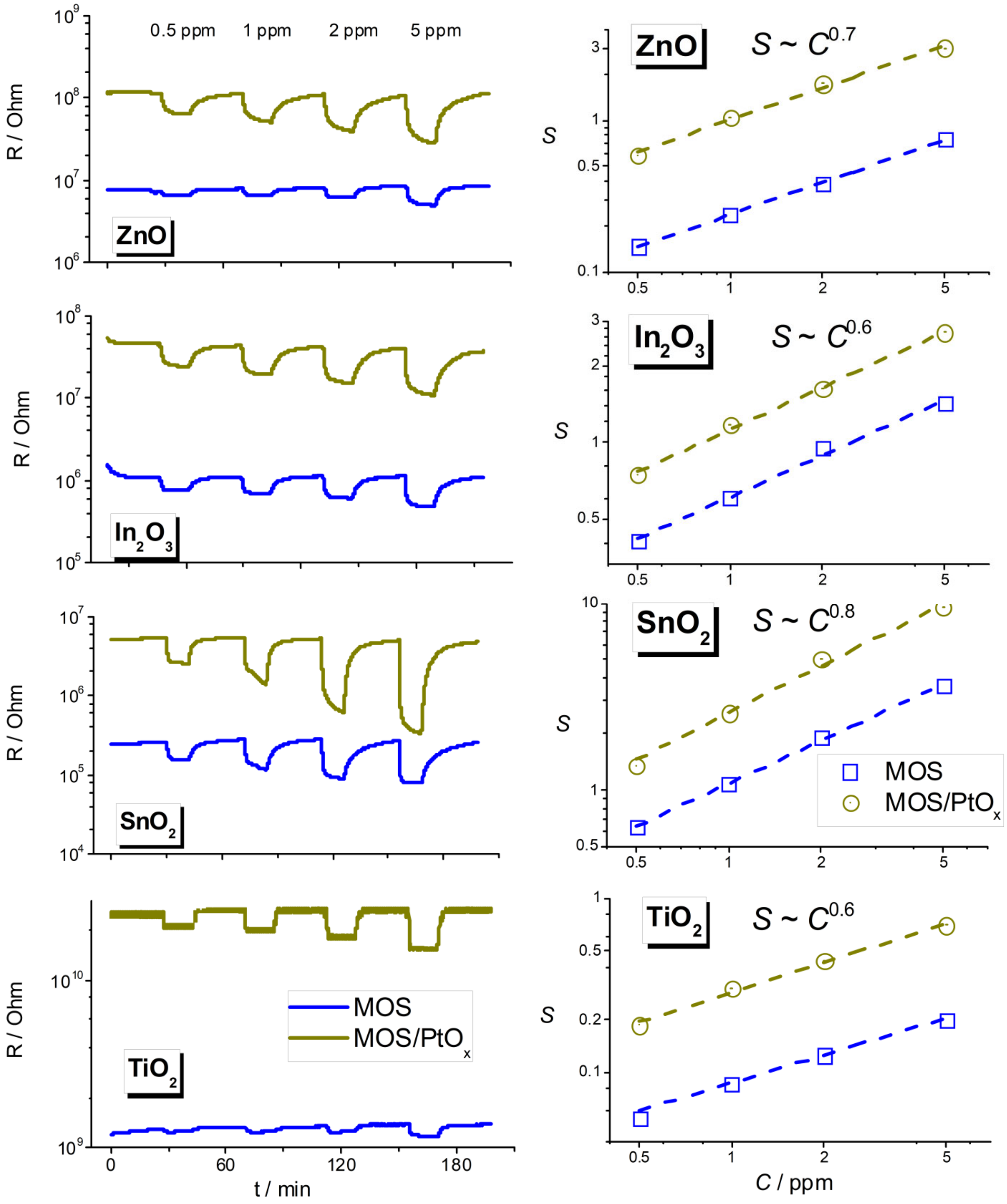

(a) **(b)**

Figure S4. Dynamic response of pristine and PtO_x-modified ZnO, In_2O_3, SnO_2, and TiO_2 to 0.5–5 ppm benzene at 220 °C (***a***), sensor signals in relation to benzene concentration at 220 °C (***b***).
